# A Second New Species of Ice Crawlers from China (Insecta: Grylloblattodea), with Thorax Evolution and the Prediction of Potential Distribution

**DOI:** 10.1371/journal.pone.0012850

**Published:** 2010-09-22

**Authors:** Ming Bai, Karl Jarvis, Shu-Yong Wang, Ke-Qing Song, Yan-Ping Wang, Zhi-Liang Wang, Wen-Zhu Li, Wei Wang, Xing-Ke Yang

**Affiliations:** 1 Key Laboratory of Zoological Systematics and Evolution, Institute of Zoology, Chinese Academy of Sciences, Beijing, People's Republic of China; 2 Northern Arizona University, Flagstaff, Arizona, United States of America; 3 Foreign Economic Cooperation Office, Ministry of Environmental Protection, Beijing, People's Republic of China; American Museum of Natural History, United States of America

## Abstract

Modern grylloblattids are one of the least diverse of the modern insect orders. The thorax changes in morphology might be associated with the changes of the function of the forelegs, wing loss, changes in behavior and adaptation to habitat. As temperature is the main barrier for migration of modern grylloblattids, the range of each species is extremely limited. The potential distribution areas of grylloblattids remain unclear. A second new species of ice crawlers (Insecta: Grylloblattodea), *Grylloblattella cheni* Bai, Wang *et* Yang **sp. nov.**, is described from China. The distribution map and key to species of *Grylloblattella* are given. A comparison of the thorax of extant and extinct Grylloblattodea is presented, with an emphasis on the pronotum using geometric morphometric analysis, which may reflect thorax adaptation and the evolution of Grylloblattodea. Potential global distribution of grylloblattids is inferred. Highly diversified pronota of extinct Grylloblattodea may reflect diverse habitats and niches. The relatively homogeneous pronota of modern grylloblattids might be explained by two hypotheses: synapomorphy or convergent evolution. Most fossils of Grylloblattodea contain an obviously longer meso- and metathorax than prothorax. The length of the meso- and metathorax of modern grylloblattids is normally shorter than the prothorax. This may be associated with the wing loss, which is accompanied by muscle reduction and changes to the thoracic skeleton system. Threats to grylloblattids and several conservation comments are also provided.

## Introduction

Modern grylloblattids (also known as ice bugs, ice crawlers, and rock crawlers), all occur northward of ∼35° latitude in cool-temperate areas of the United States, Canada, Russia, Japan, Korea and China, and they are restricted to cold and extreme habitats that are difficult to access. They are one of the least diverse of the modern insect orders, consisting of 29 species, including a new species described below. All of the known extant species, which belong to the family Grylloblattidae and 5 genera, *Galloisiana*, *Grylloblattina*, *Grylloblattella*, *Namkungia* and *Grylloblatta*. Ice crawlers can be considered as “living fossils” with presently relict distributions [1], [2]. Grylloblattids are generally found on north-facing talus slopes, snow patches near forest at high elevations (1500–3000 m), in caves with permanent ice at low elevations (300–1000 m) [3], [4], and some *Grylloblattina* are from 5 m–300 m, much lower than most other grylloblattids [5]. They live on and in soil, in caves, and beneath stones and in crevices of mountainous regions. They are principally carrion feeders on other insects, though they will consume plant material, fungus, and detritus [6].

**Figure 1:**
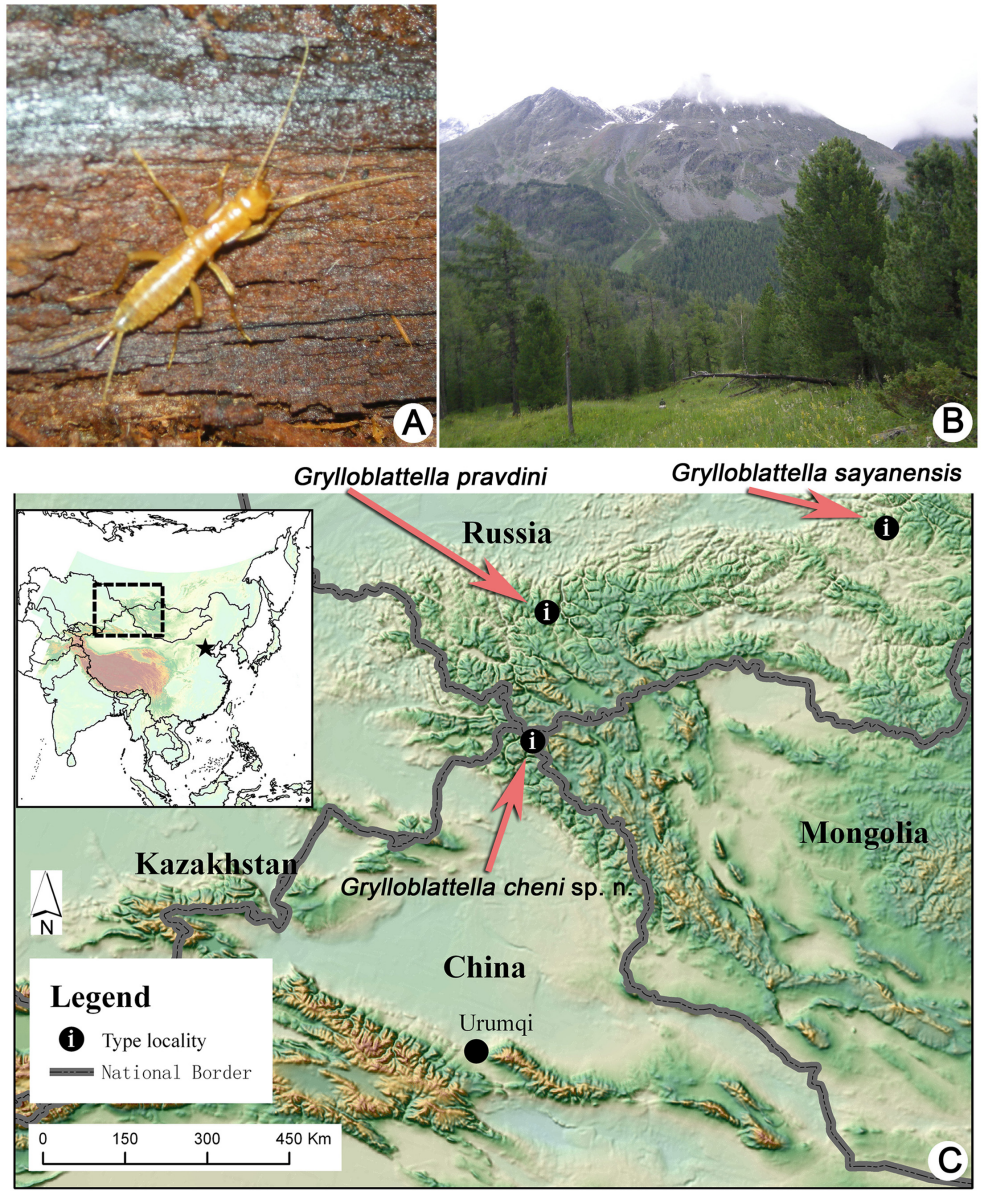
*Grylloblattella cheni* Bai, Wang *et* Yang sp. nov. (A) Female. (B) Habitat. (C) Type localities of all known three species of *Grylloblattella*.

Grylloblattids are extremely rare in China. Mr. Shu-Yong Wang collected the first grylloblattid from China, which was one male *Galloisiana sinensis* Wang, 1987 [7] specimen from Mt. Changbaishan, Jilin in 1986. Over 20 years later, Mr. Ke-Qing Song collected the second grylloblattid from China, which is one female *Grylloblattella cheni* **sp. nov.** in Akekule Lake (White Lake), Xinjiang, China. The inclusion of this species to *Grylloblattella* expands the genus to 3 species, the other two species being found in western to central Siberia, Russia: *G. pravdini* in the Altai Mountains and *G. sayanensis* in the Sayan Mountains (Fig. 1C).

Modern grylloblattids are 14–34 mm long, wingless, pale, and either nocturnal or cavernicolous. Adults have long cerci with 5–10 segments, and females have a sword-shaped ovipositor similar in shape to that of katydids (Orthoptera: Tettigoniidae). The single extant family can be contrasted with 46 families described from the fossil record, which extend to the Late Carboniferous [8], [9], [10]. The morphology of grylloblattodeans was stable with only minor changes during the ∼300 Million years of evolution, except thorax variations, which are the most significant difference between extant and extinct members of Grylloblattodea. The thorax, which contains the muscles of the legs and wings, had changed in some degree during the evolution of Grylloblattodea. This might be associated with the changes of the function of the forelegs, wing loss, changes in behavior and adaptation to habitat. Here we present a comparison of the thorax of extant and extinct Grylloblattodea, with an emphasis on the pronotum using geometric morphometric analysis, which may reflect thorax adaptation and the evolution of Grylloblattodea.

Few entomologists have ever collected these unique insects, and little is known about their life history and biology. However, the potential distribution areas of the world are relative broad according our prediction in this study. Industrial development, human activities and global warming may threaten unknown and undiscovered grylloblattids. Several conservation comments are also provided.

## Results

### Taxonomy

Genus *Grylloblattella* Storozhenko, 1988 [11]

*Diagnosis*: *Grylloblattella* can be distinguished from other genus in Grylloblattidae as follows: eyes black; antennae 27–38-segmented, epicranial suture not reaching the circumantennal suture; lacinia with one or two teeth; posterior margin of pronotum incurved, without marginal area; tarsal pulvilli visible; cerci 9–10-segmented; supra-anal plate of male symmetrical, project on the posterior margin with broadly rounded or truncate tip.

**Figure 2:**
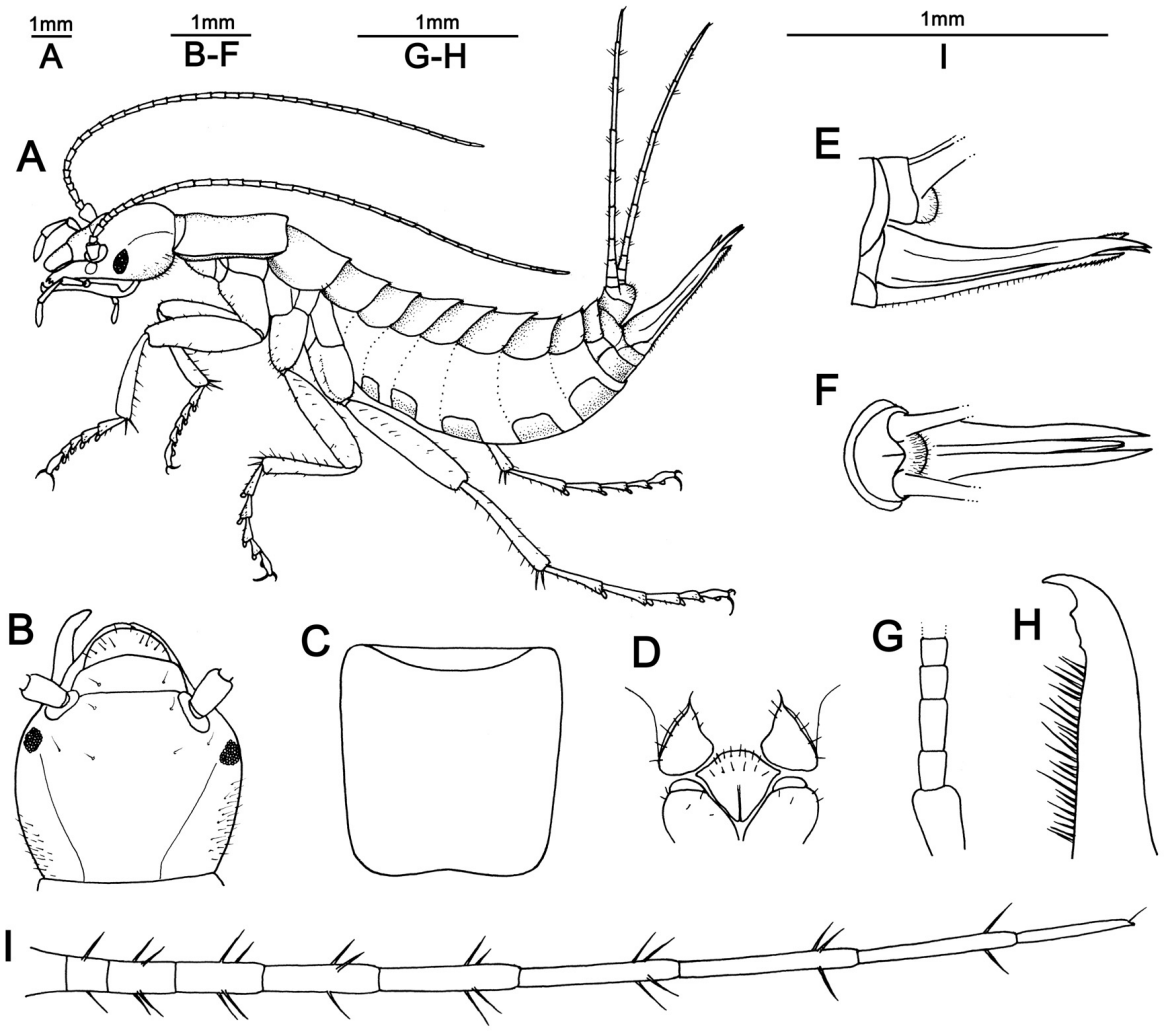
*Grylloblattella cheni* Bai, Wang *et* Yang sp. nov., female. (A) Habitus, lateral view. (B) Head, dorsal view. (C) Pronotum, dorsal view. (D) Cervical sclerites and eusterna of prothorax, ventral view. (E) Ovipositor, lateral view. (F) Ovipositor, dorsal view. (G) Basal antennomeres, left. (H) Lacinia with two preapical teeth, left. (I) Cercus, left.

*Grylloblattella cheni* Bai, Wang *et* Yang **sp. nov.** (Figs 1A, 2A–I)

urn:lsid:zoobank.org:act:E0375431-6D0E-4949-B0A0-EA99A5927104

*Holotype*: Female, CHINA, Xinjiang Province, Buerjin County, Kanas Nature Reserve, 8 km west to Akekule Lake (White Lake), north of Kanas Lake, south-east of Mt. Youyifeng (Friendship Peak), N49.04173°, E87.49166°, 1750 m, raining, 2009-VII-24; collected by Ke-Qing SONG; deposited in the collection of the Key Laboratory of Zoological Systematics and Evolution, Institute of Zoology, Chinese Academy of Sciences, People's Republic of China.

*Etymology*: This species is named in honor of Prof. Sicien Chen (Shixiang Chen), Fellow of Chinese Academy of Sciences, former PI for the Group of Morphology and Evolution of Coleoptera, Institute of Zoology, Chinese Academy of Sciences, Beijing, China. Prof. Chen was the founder and former director of the Institute of Zoology, Chinese Academy of Sciences, and he made great contributions to entomological research of China.

*Diagnosis*: This new species can be attribute to *Grylloblattella* as follows: eyes black, epicranial suture not reaching the circumantennal suture, posterior margin of pronotum incurved, and tarsal pulvilli visible. Additionally, it can be distinguished from other species in Grylloblattidae as follows: antennae 38-segmented, cervical sclerites with five setae on each of the two lateral margins, lacinia with two teeth, and cerci 10-segmented. The key to species of *Grylloblattella* is given (Table 1).

**Table 1:** Key to species of *Grylloblattella*.

1	Antennae 27–29-segmented, cerci 9-segmented, not attaining the distal end of the 7th cercomere…………………………………..……….……2
1′	Antennae 38-segmented, cerci 10-segmented, not attaining the distal end of the 6th cercomere……………*G. cheni* Bai, Wang *et* Yang **sp. nov.**
2(1)	Cervical sclerites with 2 setae on each of the two lateral margins, basisternum oviform, cerci 6.2–6.3 mm in length…..…………*G. sayanensis* Storozhenko, 1996 [12]
2′	cervical sclerites with five setae on each of the two lateral margins, basisternum triangular, cerci 4.9 mm in length………*G. pravdini* (Storozhenko *et* Oliger, 1984) [13]

*Description*: *Female (holotype)*. Total body length 14.0 mm (measured from anterior margin of labrum to posterior margin of 10th abdominal segment) (Fig. 2A). Body colored heavy orange-brown on head and thorax, lighter in color on abdominal segments, and covered with numerous short hairs (Fig. 1A).

Head attached obliquely to pronotum (Fig. 2B). Cranium wider than long (length 2.2 mm, width 2.8 mm), with six setae on each lateral margin, two setae around the antennal socket, two setae near eye, three on the posterior of each side and no seta on the middle; epicranial suture distinctly Y-shaped, not reaching the circumantennal suture, and a pair of parietal sutures extending from the occipital foramen over to the vertex. Clypeus 2.8 times wider than long, projected on its anterior middle, with distinct clypeal suture. Lacinia with two teeth (Fig. 2H). Eyes black, elongated oval in shape (Fig. 2B). Antennae filiform, composed of 38 antennomeres, the 3rd segment 1.5 times as long as the 2nd (Fig. 2G).

Pronotum 1.1 times as long as wide, slightly concave in the posterior part, with a long median suture, some hairs on the anterior and lateral margins (Fig. 2C). Mesonotum slightly concave in the posterior part, with a long median suture. Metanotum with two setae on its mid-lateral side and two longitudinal short sutures in its anterior part.

Cervical sclerites about 1.3 times as long as wide, triangular, elongated anteriorly, with five setae on each of the two lateral margins and small setae on its posterior part (Fig. 2D). Basisternum of the prothorax 1.2 times as long as wide, triangular, expanded in the anterior part, with many scattered hairs. Katepisterna of the prothorax inclined, triangular in shape, situated near the posterior part of the cervical sclerite. Trochantin of the prothorax crescent shaped, with two setae on its distal part.

Legs elongate. Coxa with scattered setae and distinct ribs on the ventral part. Profemur with one row of setae on inner margin of ventral side, no seta on lateral side of profemur; meso- and metafemur with many scattered setae. Protibia with setae on ventral side and seta on lateral side; meso- and metatibia with many scattered setae; two large spines on the apical part of all tibia. Two setae each on the apical part of the 2nd and 3rd tarsi, white pulvilli and many long hairs on all tarsi, and two strong tarsal claws without teeth. Protarsus relative length of each segment (base to apex) 13∶8∶7∶6∶11. Mesotarsus relative length of each segment (base to apex) 16∶11∶7∶5∶10. Metatarsus relative length of each segment (base to apex) 19∶11∶8∶4∶10.

Abdominal tergites with lateral margin flexed to the posterior, 10-segmented, with one seta each on the posterior margin of the 1st to 8th tergites, one seta each on the mid-lateral side of the 2nd to 7th tergites. Abdominal sternites with lateral margin flexed to the posterior, without setae.

Cercomeres ten (length 7.2 mm), cylindrical, with one ring pattern of setae on the distal part of all cercomeres except the first and terminal one (Fig. 2I). Relative length of each segment (base to apex) 3∶3∶5∶8∶10∶12∶14∶16∶15∶10. 1st cercomere without seta; 2nd cercomere with 2 setae; 3rd cercomere with four setae; 4th cercomere with five setae; 5th cercomere with four setae; 6th and 7th cercomere with four setae each; 8th and 9th cercomere with three setae; 10th cercomere with one seta.

Ovipositor situated in the ventral part of the 8th and 9th abdominal segments, symmetrical, not attaining the distal end of the 6th cercomere; gonoplac (4.1 mm) longer than the others, with numerous setae on the dorsal part; gonangulum situated between the 8th sternite and 1st gonapophysis, asymmetrical “/ \”-shaped, slightly pointed; 1st gonapophysis (length 3.7 mm) slightly curved medially, with numerous bent hairs on the lower posterior part, adjoining gonangulum, and vertically emarginated mid-anterior part (Figs 2E–F).

*Biological Notes*: The species is known only from primary boreal coniferous forest (taiga), near Lake or river (Fig. 1B). The specimen collected was found under the bark of fallen dead tree, which near snow line (about 2 km away) in summer and could be covered by over 1 meter deep snow in winter. The temperature of type locality is from 0∼10°C in summer and −30∼0°C in winter.

*Geographic Distribution*: This species is known from the type locality near Akekule Lake (White Lake) and north of Kanas Lake, Kanas Nature Reserve, Buerjin County, Xinjiang Province, China (Fig. 1C).

### Variations of pronotum morphology in Grylloblattodea

Geometric morphometrics can be used to determine shape differences, and the resulting phenograms from Procrustes distances can effectively indicate phenetic relationships, summarizing overall patterns of similarity [14]. Compared with other characters, the pronotum shows relatively high diversity in Grylloblattodea. We performed a morphometric analysis of the pronotum morphology of extant and extinct Grylloblattodea. This morphometric analysis allowed us to evaluate the similarity of the fossil pronotum to contemporary pronotum.

**Figure 3:**
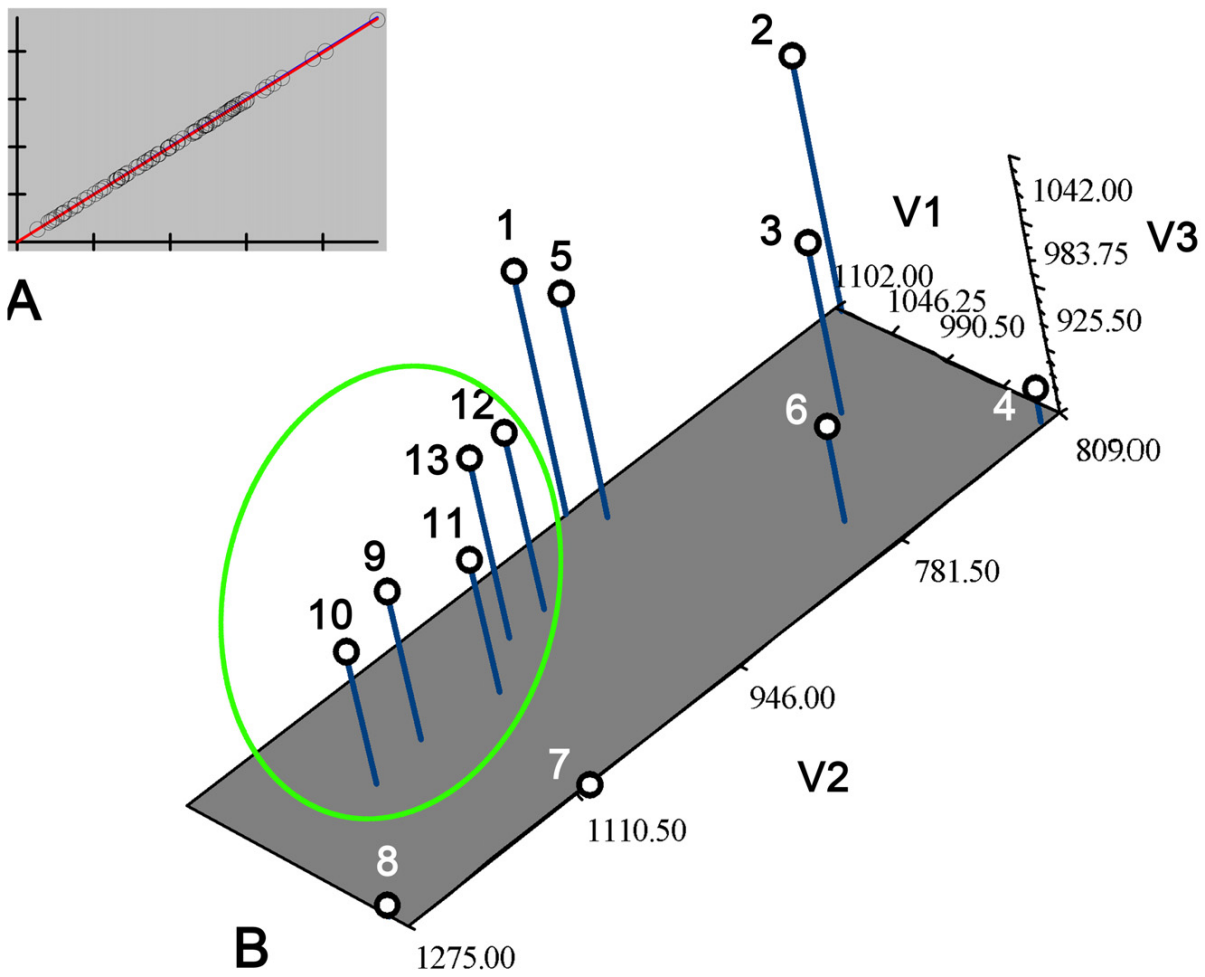
Pronotum shape variation test and shape differences of 13 grylloblattids genera. (A) Shape variation test tps-SMALL 1.2. (B) Ordination of the 13 grylloblattids genera means along the three canonical varieties axes based on the Procrustes distance matrix. (1) *Blattogryllulus*; (2) *Parasheimia*; (3) *Plesioblattogryllus*; (4) *Sojanorapbidia*; (5) *Sylvamicropteron*; (6) *Sylvonympha*; (7) *Tataronympha*; (8) *Tillyardembia*; (9) *Galloisiana*; (10) *Grylloblatta*; (11) *Grylloblattella*; (12) *Grylloblattina*; (13) *Namkungia*. Green circle includes the extant 5 grylloblattids genera.

There were 50 equidistant semilandmarks chosen on the outline of the pronotum (tps-DIG 2.05, curve). The consensus configuration for each genus was determined and the images of each species of the genus ‘unwarped’ so that the semilandmarks coincided with their positions in the consensus configuration. All the species in a single genus were then superimposed onto one another. Analyses of the data set using tps-SMALL indicated that an excellent correlation between the tangent and the shape space existed (Fig. 3A). The correlation (uncentred) between the tangent space, Y, regressed onto Procrustes distance (geodesic distances in radians) was 0.999993. There was little doubt on the basis of the result from tps-SMALL, which supported the hypothesis that genus within a taxon such as a family can be analyzed by geometric morphometric methods since the results from the statistical test performed by tps-SMALL proved the acceptability of the data set for further statistical analysis [14].

**Figure 4:**
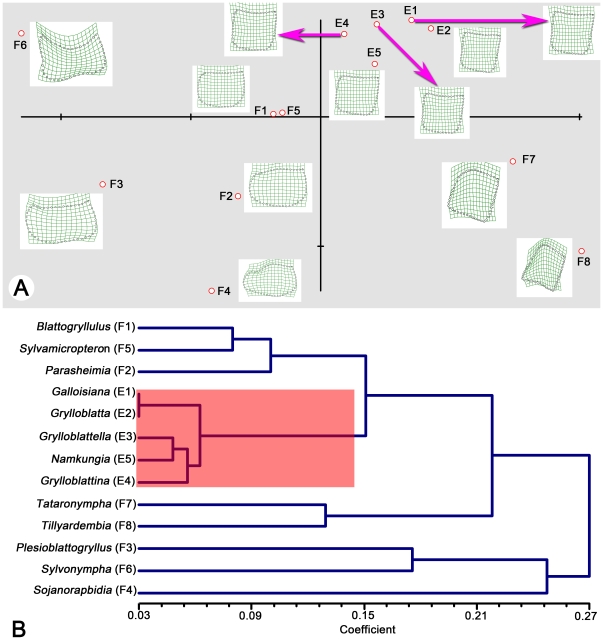
Pronotum shape differences of 13 grylloblattids genera. (A) Relative warps computed from the data set, plotted against one another to indicate positions of the relationships among genera relative to one another and to the reference configuration (situated at the origin). The shape changes of different families implied by variation along the first two relative warp axes. Shape changes are shown as deformations of the GLS reference, using thin-plate splines. (B) Phenetic tree (UPGMA), the trees compiled using NTSYS-pc based on Procrustes distances among the genera.

The first two relative warps of the semilandmarks accounted for 81.85% of the variation among the genera. These were computed by a singular-value decomposition of the weight matrix [15]. The first two relative warps were plotted to indicate variation along the two axes (Fig. 4A). The shape changes of different genera implied by variation along the first two relative warp axes and shape changes were shown as deformations of the GLS reference, using thin-plate splines (Fig. 4A). The spline of each genus showed the deformation of the semilandmarks in comparison to that of the reference. The five modern genera are in the First Quadrant and other eight fossil genera are in the other Quadrants.

The 13 grylloblattodean genera means were plotted along the three canonical varieties axes based on the Procrustes distance matrix (Fig. 3B). UPGMA phenogram [16] of the pronotum of the 13 studied genera based on Procrustes distance matrix are presented in Figure 4B. The results indicate a good correlation between the scatter plots (Figs 3B, 4A) and the phenograms (produced from the Procrustes distances) (Fig. 4B). Genera clustering together in the phenograms are closely situated on the scatter plot of the first two relative warps.

## Discussion

### Phylogenetic relationships among fossil and extant grylloblattodeans

The relationships between grylloblattodean fossil taxa and the modern grylloblattids remain unclear because most fossils are based on isolated wings or have poorly preserved body features. Wheeler *et al.* [17] proposed that the modern Grylloblattidae were the sister group of the Dermaptera, being together the sister group of (Phasmida + Orthoptera), and that the (Plecoptera + Embioptera) were not directly related to this clade. Beutel and Gorb [18] claimed that the Grylloblattidae were the sister group of the clade {Phasmatodea + [Mantodea + (Isoptera + Blattodea)]}. Grimaldi [19] considered that the extant and fossil ‘Grylloblattodea’ fell in an unresolved polytomy with the majority of the other ‘polyneopteran’ orders. Molecular phylogenies from Terry and Whiting [20] and Cameron, Barker and Whiting [21] indicated that Grylloblattodea could be the sister group of the recently discovered order Mantophasmatodea, and altogether could be the sister group of the Dictyoptera. Grimaldi and Engel [22] also considered that the Palaeozoic and Mesozoic alate ‘grylloblattids’ could represent a stem group of both apterous Grylloblattodea and Mantophasmatodea. Lastly, even if Rasnitsyn [23], [24] listed several similarities between *Blattogryllus* and the extant Grylloblattidae, the potential synapomorphies of this last group with the fossil ‘Grylloblattodea’ are not clear. We present possible relationships of all modern grylloblattids and eight extinct genera based on the morphology of pronotum, which is a highly diverse character in Grylloblattodea. This result suggests a new way to infer the phylogeny of fossil taxa and modern grylloblattids, which bridge the huge gap between extremely diverse extinct winged taxa and rare extant wingless grylloblattids.

Little is known of extant grylloblattid genus and species phylogeny. Storozhenko [25] proposed a phylogeny of four extant grylloblattid genera, *Grylloblatta*, *Galloisiana*, *Grylloblattina*, and *Grylloblattella*, based on an intuitive analysis of ten morphological and two habitat characters. A single character supports the monophyly of the Asian genera (presence of four to eight setae on the edges of the cervical sclerites, as opposed to none in *Grylloblatta*), rendering the Asian genera as sister group to *Grylloblatta* [25]. Among the Asian genera, Storozhenko places *Grylloblattina* as sister to *Galloisiana*+*Grylloblattella*, supported by the narrow elongated condition of the right coxopodite of abdominal segment IX of the male in *Galloisiana* and *Grylloblattella* rather than a short thickened one in *Grylloblattella*. Jarvis and Whiting [1] presents the first-ever formal phylogenetic hypothesis of modern grylloblattid genera and species based on molecular evidence. The topology confirms the monophyly of the three genera included in the analysis: *Grylloblatta*, *Galloisiana*, and *Grylloblattina*. The analysis indicates that the eastern Asian genus *Galloisiana* is sister to a monophyletic group of the east Siberian *Grylloblattina* and the North American *Grylloblatta*. Our result not only confirms the phylogenetic hypothesis of *Grylloblatta*, *Galloisiana*, and *Grylloblattina* from Jarvis and Whiting [1], but also presents the relationships of all known modern grylloblattid genera.

### Thorax evolution in Grylloblattodea

The thorax must have evolved early in the phylogenetic history of the Hexapoda as a locomotor section of the body through the specialization of its appendages for quicker movement [26]. The evolution of thorax morphology may be correlated with movement functions involved in walking and flying.

Our results indicate that there was much higher diversity in the pronotum of fossil species than in modern grylloblattids. This may be due to the totally different habitats in extant and extinct species. The food habits of the early grylloblattodeans, such as pollen feeding, predation, etc., were very diverse, according to the fossil record. For example, *Plesioblattogryllus magnificus* from Middle Jurassic with the movable structures composed of the fore tarsal claws, the most apical tarsomeres, and very strong mandibles with a sharp pointed apical tooth is considered as an active hunter [27]. Winged members of Grylloblattodea might have lived in a relatively warm environment and a variety of habitat types in the Paleozoic and Mesozoic Era. Highly diversified pronota might reflect a diverse habitats and niches. Modern grylloblattids probably became adapted to live under rocks or hidden in moss from cold areas. The lack of pronotal variation in modern grylloblattids might be explained by two hypotheses: synapomorphy or convergent evolution. The first hypothesis proposes a single clade supported by the character of a nearly rectangular pronotum, which has survived after the extinction of other grylloblattodean taxa. The second hypothesis proposes that the pronotum of the ancestors of modern grylloblattids were different in shape. After the extinction of most grylloblattodeans, the remaining species lived in similar environments, which drove convergent evolution in pronotum shape.

Insects are the only invertebrate animals which have wings. Flight conferred an increased ability to access resources, locate mates and escape predators [28], and has undoubtedly contributed to the success of insects. Despite the obvious advantages of flight, this dispersal capacity has been lost repeatedly [29], [30] in nearly all winged orders [31]. The loss of flight, typically due to a reduction in wing length, has been attributed to the high energy expenditure required in the production and maintenance of flight apparatus, at the expense of other life-history traits [32]. Low temperature may be the key factor for the wing loss [33]. Wings are only found on the mesothorax and metathorax, and the prothorax never bears wings in extant insects. Mesothorax and metathorax of grylloblattids maintain very low variation in shape during ∼300 Million years evolution, which bears the wings in Paleozoic and Mesozoic Era. Most grylloblattid fossils contain an obviously longer meso- and metathorax than prothorax. The length of the meso- and metathorax of modern grylloblattids is normally shorter than the prothorax. This may be associated with the wing loss, which is accompanied by muscle reduction and changes to the thoracic skeleton system.

### Threats to grylloblattids and potential distribution areas

As temperature is the main barriers for migration of modern grylloblattids, the distribution area of one species is very limited. Migration among populations is almost certainly severely limited or non-existent in current conditions due to grylloblattids' habitat specificity, limited geographic range of populations, and winglessness [4]. The warming of the planet since the last glaciation, compounded by human-induced global warming in recent years is causing the rapid loss of glaciers and ice sheets [34], [35], [36], [37]. In the next few decades, the rate at which glaciers are melting is expected to increase by two to four times from their already high rate, largely due to anthropogenic causes [34]. Grylloblattids' dependence on glacial margin habitats means that global warming is a direct threat to their future. Resilience of grylloblattid habitat cannot be expected without significant changes in factors linked to glacial decline.

**Figure 5:**
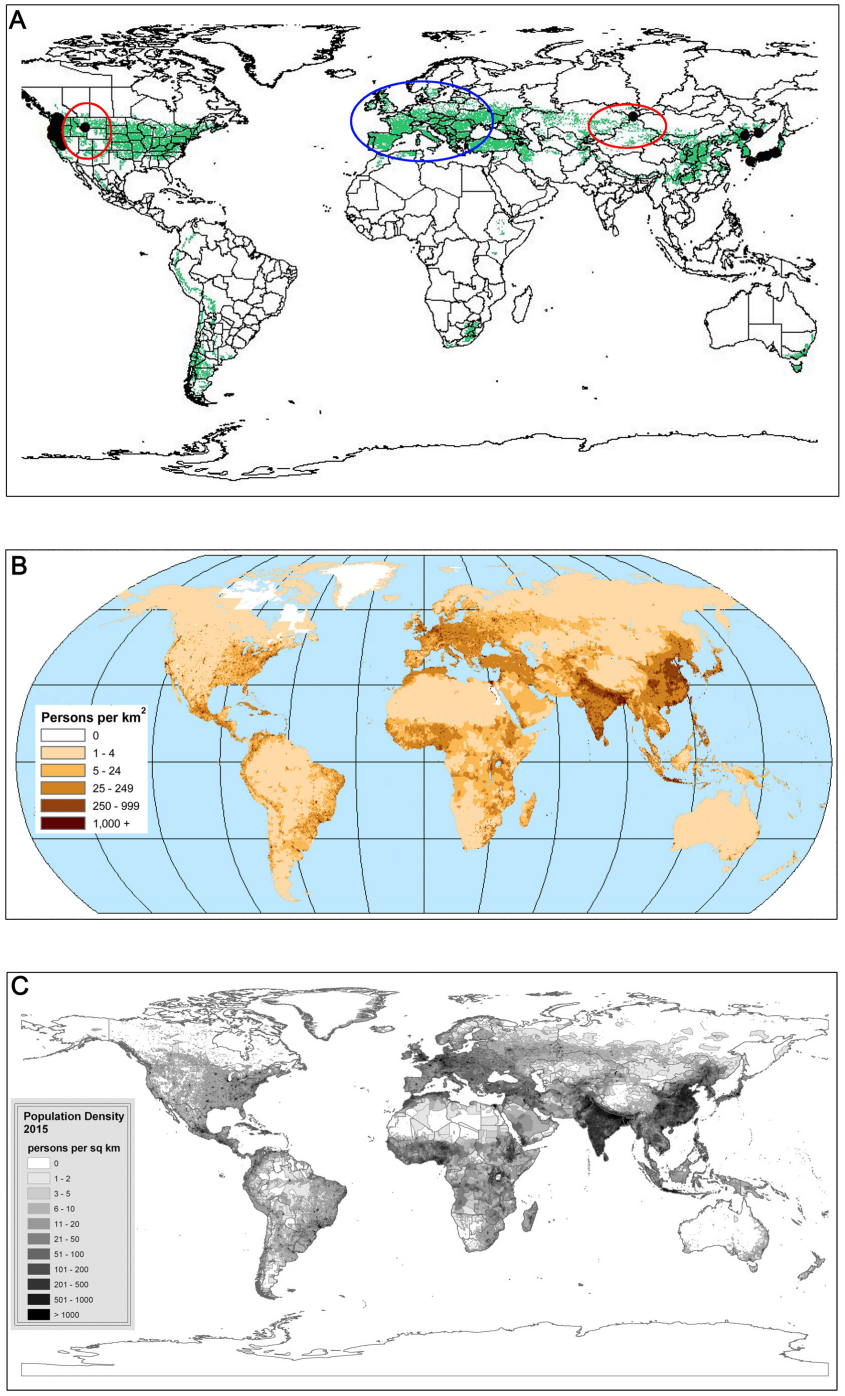
The comparison of the prediction map of grylloblattids and the map of Population Density of the world. (A) Prediction of potential distribution areas of grylloblattids; black dots  =  selected known localities, green areas  =  potential distribution areas, red circles  =  the most potential areas, blue circle  =  the least potential areas. (B) Population Density of the world in 2000 (after CIESIN and CIAT 2005). (C) Population Density of the world in 2015 (after CIESIN, FAO and CIAT 2005).

Another potentially significant threat to grylloblattids is development of their habitat. The known localities (Fig. 5A, black dots) of grylloblattids are very remote areas, and the potential distribution areas we inferred have environments similar to those of known distribution localities. As grylloblattids can be found in two typical place: high mountain and ice cave which might from lowland or mountain, we run the two prediction analyses. Firstly, all occurrence locations with coordinates were selected for the raw analysis, which could reflect the all possible areas (Fig. 5A). Our results show that the potential distribution of grylloblattid species are near regions populated by humans (Fig. 5A and 5B) [38]. In 2015 [39], the distribution and density of human populations be greater than in 2000 (Figs 5B–C). Human interference has caused major environmental damage in potential and actual distribution areas of grylloblattids. Based on these evidences, we propose two areas (Fig. 5A, red circles) in which grylloblattids may possibly occur. We assume that the high degree of human interference in Europe (Fig. 5A, blue circle) would greatly reduce the potential to find grylloblattids there.

**Figure 6:**
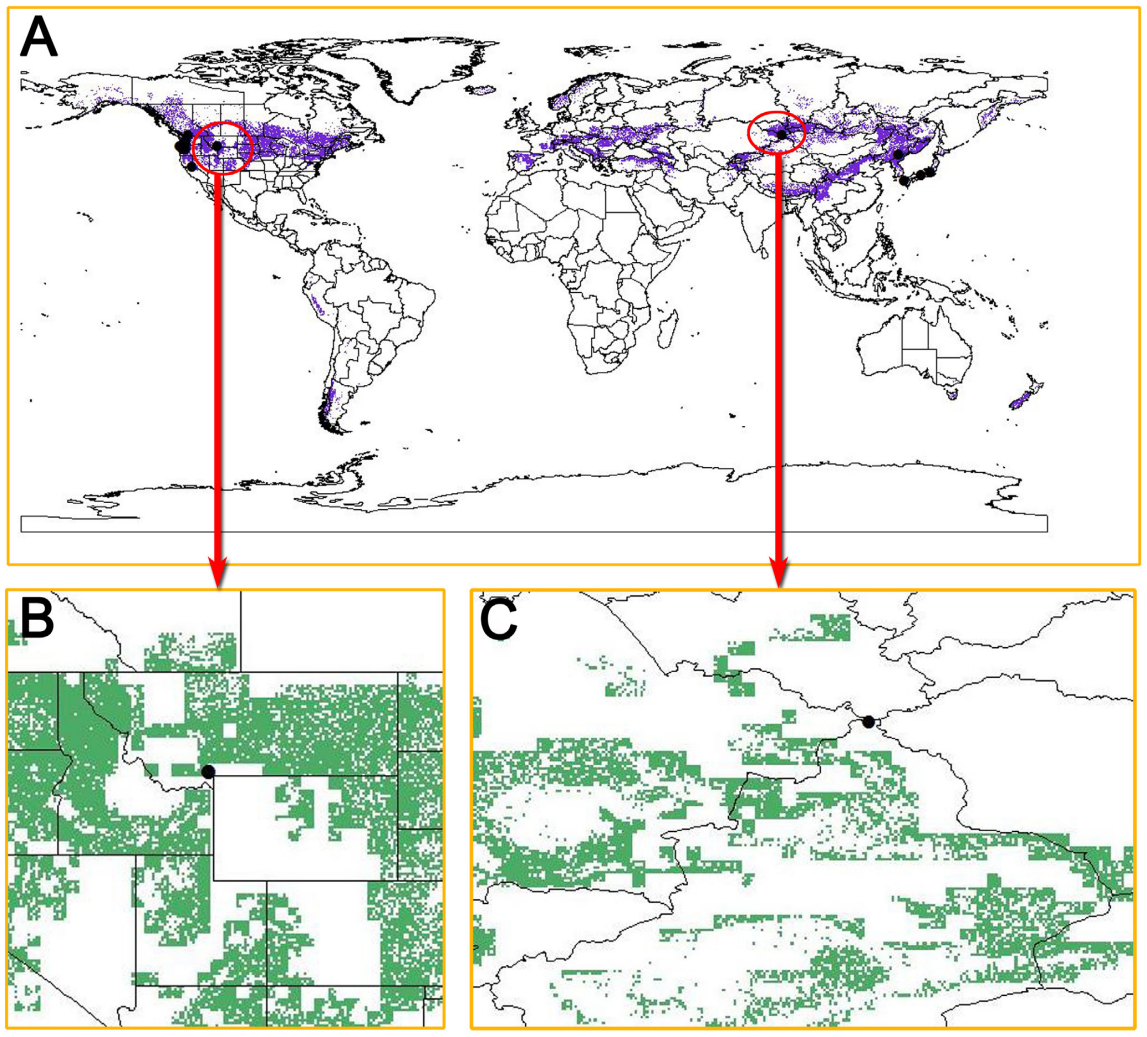
Prediction map of grylloblattids based on the 19 coordinates of terrestrial ice crawlers. (A) Prediction of potential distribution areas of grylloblattids. (B) Areas in the US with the most potential. (C) Areas in Asia with the most potential areas. Black dots  =  selected known localities, purple areas (in A)  =  potential distribution areas, green areas (in B or C)  =  potential distribution areas, red circles (in A)  =  areas with the most potential.

Secondly, only high mountain data were used in the specific analysis for the prediction of high mountain grylloblattids (Fig. 6A). This map is almost same to the first prediction analysis (see Fig 5A), but a little bit shrinking in the prediction areas. The amplificatory map of two areas (Fig. 6A, red circles) indicates where grylloblattids may possibly occur.

The purpose of our prediction research is not to explain the distribution of grylloblattids, but to predict where potential areas are. The reason why many grylloblattids have been found in Japan (see Fig. 5A) may be due to high human population density close to pristine grylloblattid habitat. For example, it is doubtful that there are many more new species that could be found in Japan because of how much work has been done on grylloblattids there already. In the recent past, no new species have been found in Japan, but in South Korea and China there have been several new species discovered [2], [40]. We expect that more undiscovered species could exist in many of the other predicted areas shaded green, but that it depends on 1) the accessibility of relatively undisturbed grylloblattid habitat, and 2) the number of people interested and knowledgeable enough to search for and describe new species.

The reason for the collection of *Grylloblattella cheni* Bai, Wang *et* Yang **sp. nov.** by Mr. Ke-Qing Song was an environmental assessment project on planned railway construction in Xinjiang, China. The report (unpublished report for North Xinjiang Environment Exploration Program) based on this assessment concluded that although the precise effects of railway construction on *Grylloblattella cheni* cannot be determined, there will certainly be detrimental effects on this exceptionally rare species. In order to preserve grylloblattid habitat, we suggest the railway be routed through lower elevation terrain, which would cause minimal disturbance to grylloblattids.

Potential distribution areas of grylloblattids are scattered over much wider areas than the very limited type localities (Fig. 5A). Most of these potential distribution areas are remote areas that are typically low in biodiversity. None of these areas are in the list of the well-known biodiversity hotspots, and insect surveys for conservation purposes are rarely conducted. Therefore it is imperative that more research be done in these regions in order to provide insight into the ecosystems that contain these unique organisms.

## Materials and Methods

### Nomenclatural Acts

The electronic version of this document does not represent a published work according to the International Code of Zoological Nomenclature (ICZN), and hence the nomenclatural acts contained in the electronic version are not available under that Code from the electronic edition. Therefore, a separate edition of this document was produced by a method that assures numerous identical and durable copies, and those copies were simultaneously obtainable (from the publication date noted on the first page of this article) for the purpose of providing a public and permanent scientific record, in accordance with Article 8.1 of the Code. The separate print-only edition is available on request from PLoS by sending a request to PLoS ONE, 1160 Battery Street Suite 100, San Francisco, CA 94111, USA along with a check for $10 (to cover printing and postage) payable to “Public Library of Science”.

In addition, this published work and the nomenclatural acts it contains have been registered in ZooBank, the proposed online registration system for the ICZN. The ZooBank LSIDs (Life Science Identifiers) can be resolved and the associated information viewed through any standard web browser by appending the LSID to the prefix “http://zoobank.org/”. The LSID for this publication is: urn:lsid:zoobank.org:pub:1157690F-E3D2-4684-82DB-B6872A7F4964.

### Geometric morphometric analysis

**Figure 7:**
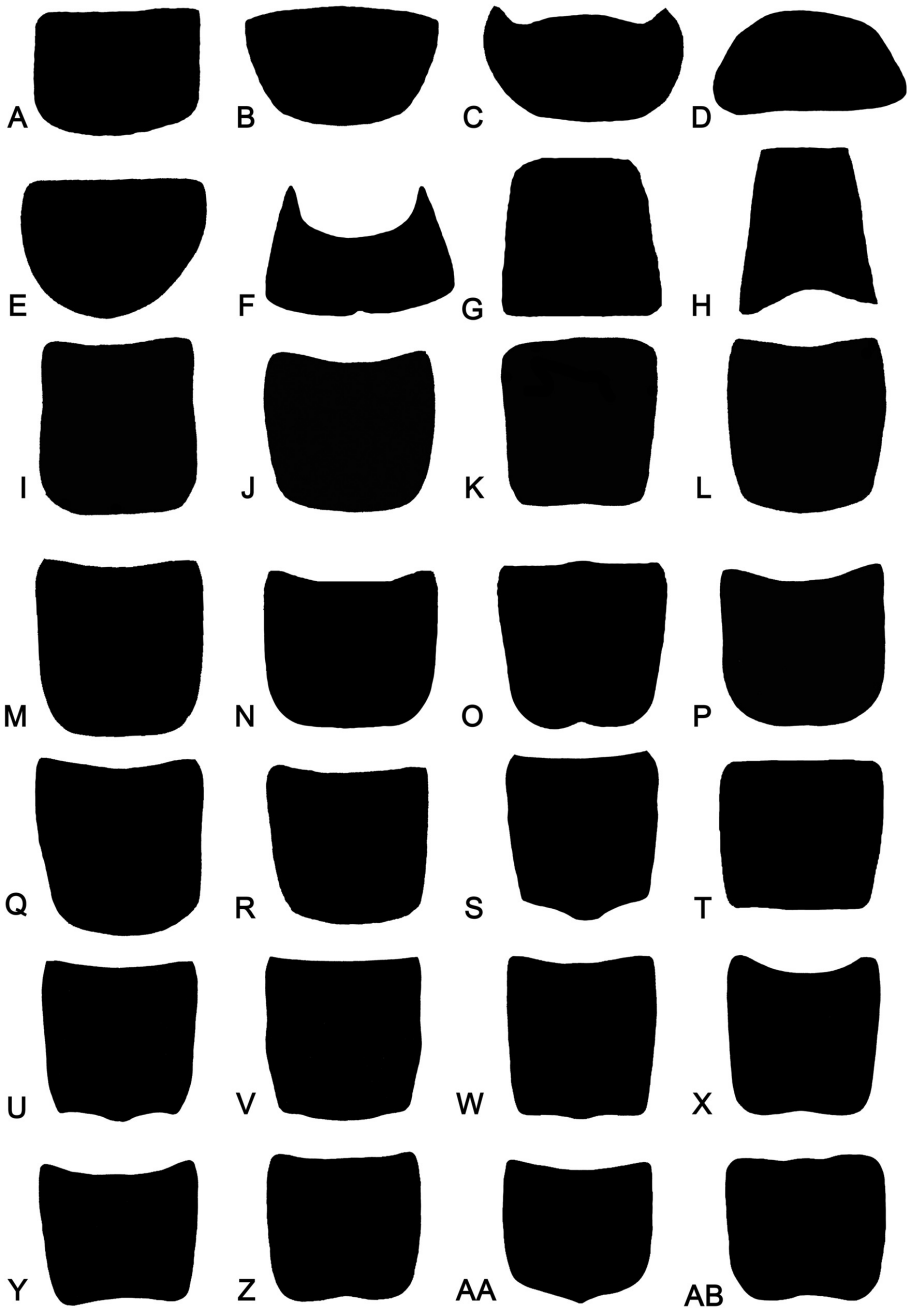
Pronotum shape of 13 grylloblattids for the geometric morphometric analysis. (A) *Blattogryllulus mongolicus* Storozhenko, 1988 (Fossil). (B) *Parasheimia truncata* Aristov, 2004 (Fossil). (C) *Plesioblattogryllus magnificus* Huang, 2008 (Fossil). (D) *Sojanorapbidia martynovae* Storozhenko *et* Novokshonov, 1994 (Fossil). (E) *Sylvamicropteron harpax* Aristov, 2004 (Fossil). (F) *Sylvonympha tshekardensis* Novokshonov *et* Pan'kov, 1999 (Fossil). (G) *Tataronympha kamensis* Aristov, Novokshonov *et* Pan'kov, 2006 (Fossil). (H) *Tillyardembia antennaeplana* Zalessky, 1938 (Fossil). (I) *Galloisiana chujoi* Gurney, 1961. (J) *G. kiyosawai* Asahina, 1959. (K) *G. kosuensis* Namkung, 1974. (L) *G. nipponensis* (Caudell *et* King, 1924). (M) *G. odaesanensis* Kim *et* Lee, 2007. (N) *G. olgae* Vrsansky *et* Storozhenko, 2001. (O) *G. sinensis* Wang, 1987. (P) *G. ussuriensis* Storozhenko, 1988. (Q) *G. yezoensis* Asahina, 1961. (R) *G. yuasai* Asahina, 1959. (S) *Grylloblatta barberi* Caudell, 1924. (T) *G. campodeiformis* Walker, 1914. (U) *G. chandleri* Kamp, 1963. (V) *G. gurneyi* Kamp, 1963. (W) *G. sculleni* Gurney 1937. (X) *Grylloblattella cheni* Bai, Wang *et* Yang **sp. nov.** (Y) *G. pravdini* (Storozhenko *et* Oliger, 1984). (Z) *G. sayanensis* Storozhenko, 1996. (AA) *Grylloblattina djakonovi* Bey-Bienko, 1951. (AB) *Namkungia biryongensis* (Namkung, 1974).

Digital images of grylloblattodean pronota for the morphometric analysis were from the references, edited and enhanced by Photoshop (Version 9.0). Eight fossil species belonging to eight genera and 20 extant species belonging to five genera were included in the analysis (Table 2). As most fossils of Grylloblattodea are based on isolated wings or have poorly preserved body features, only eight genera with complete pronota in the fossils are involved included in the analysis (Fig. 7).

**Table 2:** Geometric morphometric analysis of grylloblattodean pronota represented by eight fossil species and 20 extant species.

**Extinct**	*Blattogryllulus mongolicus* Storozhenko, 1988 [41]
	*Parasheimia truncata* Aristov, 2004 [42]
	*Plesioblattogryllus magnificus* Huang, Nel *et* Petrulevicius, 2008 [27]
	*Sojanorapbidia martynovae* Storozhenko *et* Novokshonov, 1994 [43]
	*Sylvamicropteron harpax* Aristov, 2004 [42]
	*Sylvonympha tshekardensis* Novokshonov *et* Pan'kov, 1999 [44]
	*Tataronympha kamensis* Aristov, Novokshonov *et* Pan'kov, 2006 [45]
	*Tillyardembia antennaeplana* Zalessky, 1938 [46]
**Extant**	*Galloisiana chujoi* Gurney, 1961 [47]
	*G. kiyosawai* Asahina, 1959 [48]
	*G. kosuensis* Namkung, 1974 [49]
	*G. nipponensis* (Caudell *et* King, 1924) [50]
	*G. odaesanensis* Kim *et* Lee, 2007 [51]
	*G. olgae* Vrsansky *et* Storozhenko, 2001 [2]
	*G. sinensis* Wang, 1987 [7]
	*G. ussuriensis* Storozhenko, 1988 [11]
	*G. yezoensis* Asahina, 1961 [52]
	*G. yuasai* Asahina, 1959 [48]
	*Grylloblatta barberi* Caudell, 1924 [53]
	*G. campodeiformis* Walker, 1914 [54]
	*G. chandleri* Kamp, 1963 [3]
	*G. gurneyi* Kamp, 1963 [3]
	*G. sculleni* Gurney 1937 [55]
	*Grylloblattella cheni* Bai, Wang *et* Yang sp. nov.
	*G. pravdini* (Storozhenko *et* Oliger, 1984) [13]
	*G. sayanensis* Storozhenko, 1996 [12]
	*Grylloblattina djakonovi* Bey-Bienko, 1951 [56]
	*Namkungia biryongensis* (Namkung, 1974) [57]

In this study, 50 semilandmarks were selected. Photographs were input to tps-UTILS 1.38 [58] and Cartesian coordinates of semilandmarks were digitized with tps-DIG 2.05 [59]. We drew a curve along the outer margin of the pronotum first. Then 50 semilandmarks were resampled by length for the curve. Semilandmark configurations were scaled, translated and rotated against the consensus configuration using the GLS Procrustes superimposition method [60]. We used tps-SMALL 1.2 [61] to test whether the observed variation in shape was sufficiently small that the distribution of points in the tangent space can be used as a good approximation of their distribution in shape space (Fig. 3A). Because shape differences between genera were studied, the average or consensus configuration of semilandmarks for each genus was computed using tps-SUPER 1.14 [62]. Orthogonal least-squares Procrustes average configurations of semilandmarks were computed using generalized orthogonal least-squares procedures. Then the new TPS file with 13 taxa was created for the following process. The coordinates were analyzed using tps-RELW 1.44 [63] to calculate eigenvalues for each principal warp (Fig. 4A). Procrustes distances (the square root of the sum of squared differences between corresponding points) between each of the genera were computed and the matrix was produced by the tps-SPLIN 1.20 [64]. The Procrustes distance matrix was subjected to UPGMA (unweighted pair group method using arithmetic averages) generated by NTSYSpc [65] to determine the phenetic relationships among genera (Fig. 4B). The most important advantage of using Procrustes distances to capture shape variation was that these distances were considered the best method for measuring shape differences among taxa [14], [66], [67], [68], [69], [70].

### Prediction of potential distribution areas of grylloblattids

Ecological niches and geographic distributional prediction of ice crawlers were modeled using the Genetic Algorithm for Rule-set Prediction (GARP) [71], implemented as DesktopGarp v.1.1.6 downloaded from http://www.nhm.ku.edu/desktopgarp/Download.html. DesktopGarp is a software package for biodiversity and ecological research, which models ecological niches of species and predicts their potential distributions [71].

The geographic potential distributions were generated with GARP through a genetic algorithm that creates a series of rules matching the species specific ecological characteristics with known location occurrences [72], [73].

The species' current distributional points and the environmental datasets designed from groups of environmental layers were entered into DesktopGarp. The environmental data layers were available through the DesktopGarp website.

The ice crawler occurrence data were obtained from the published literature, available specimen from museum or personal collections. Over hundreds of distribution data of grylloblattids were collected at first. Due to the lack of coordinates for most of them, only 42 occurrence locations with coordinates were selected for our analysis (Table S1). As 23 occurrence locations represented ice caves from lowland, which could not be very idea data resource for the prediction of potential distribution areas of none-cave-living ice crawler. We ran the prediction analysis again based on the 19 coordinates of mountain ice crawler populations, for which data from ice caves were excluded.

## Supporting Information

Table S1 (0.09 MB DOC)
