# Supplementary material for: A Second New Species of Ice Crawlers from China (Insecta: Grylloblattodea), with Thorax Evolution and the Prediction of Potential Distribution
Source: PLoS One. 2010 Sep 22;5(9):e12850. doi: 10.1371/journal.pone.0012850 (PMC2943926; doi:10.1371/journal.pone.0012850)
Supplement: Table S1 — (0.09 MB DOC) [file pone.0012850.s001.doc]

**Table S1** The list of 42 ice crawler occurrence data for the prediction analysis of potential distribution areas of grylloblattids. (UW-Burke Museum= Burke Museum of Natural History and Culture, University of Washington, USA; IZAS= Institute of Zoology, Chinese Academy of Sciences, China)

| **Genus** | **species** | **Longitude** | **Latitude** | **Data resource** | **Habitat** |
| --- | --- | --- | --- | --- | --- |
| *Grylloblatta* | *gurneyi* | -121.56 | 41.73 | Jarvis & Whiting, 2006 | ice cave |
| *Grylloblatta* | sp. | -121.7 | 41.39 | Jarvis & Whiting, 2006 | ice cave |
| *Grylloblatta* | *gurneyi* | -121.51 | 41.71 | Jarvis & Whiting, 2006 | ice cave |
| *Grylloblatta* | *gurneyi* | -121.5 | 41.72 | Jarvis & Whiting, 2006 | ice cave |
| *Grylloblatta* | *gurneyi* | -121.61 | 41.5 | Jarvis & Whiting, 2006 | ice cave |
| *Grylloblatta* | *gurneyi* | -121.69 | 41.7 | Jarvis & Whiting, 2006 | ice cave |
| *Grylloblatta* | *gurneyi* | -121.6 | 41.73 | Jarvis & Whiting, 2006 | ice cave |
| *Grylloblatta* | *gurneyi* | -121.5 | 41.73 | Jarvis & Whiting, 2006 | ice cave |
| *Grylloblatta* | *bifratrilecta* | -119.64 | 38.33 | Gurney, 1953 | mountain |
| *Grylloblatta* | sp. | -120.33 | 39.42 | Jarvis & Whiting, 2006 | mountain |
| *Grylloblatta* | *rothi* | -121.81 | 44.26 | Jarvis & Whiting, 2006 | mountain |
| *Grylloblatta* | sp. | -121.77 | 44.14 | Jarvis & Whiting, 2006 | mountain |
| *Grylloblatta* | sp. | -123.41 | 42.1 | Jarvis & Whiting, 2006 | ice cave |
| *Grylloblatta* | sp. | -122.5 | 44.48 | Jarvis & Whiting, 2006 | mountain |
| *Grylloblatta* | sp. | -121.98 | 44.43 | Jarvis & Whiting, 2006 | ice cave |
| *Grylloblatta* | *sculleni* | -123.51 | 44.5 | Jarvis & Whiting, 2006 | mountain |
| *Grylloblatta* | *chirurgica* | -122.26 | 46.07 | UW - Burke Museum | ice cave |
| *Grylloblatta* | sp. | -121.55 | 46.75 | UW - Burke Museum | mountain |
| *Grylloblatta* | sp. | -121.78 | 46.43 | UW - Burke Museum | mountain |
| *Grylloblatta* | sp. | -120.65 | 48.52 | Merrill Peterson | mountain |
| *Grylloblatta* | sp. | -121.81 | 47.02 | UW - Burke Museum | mountain |
| *Grylloblatta* | sp. | -121.8 | 48.64 | UW - Burke Museum | ice cave |
| *Grylloblatta* | *chirurgica* | -122.21 | 46.11 | Jarvis & Whiting, 2006 | ice cave |
| *Grylloblatta* | *chirurgica* | -122.24 | 46.07 | Jarvis & Whiting, 2006 | ice cave |
| *Grylloblatta* | *chirurgica* | -122.23 | 46.08 | UW - Burke Museum | ice cave |
| *Grylloblatta* | *chirurgica* | -122.24 | 46.08 | UW - Burke Museum | ice cave |
| *Grylloblatta* | *chirurgica* | -122.23 | 46.14 | UW - Burke Museum | ice cave |
| *Grylloblatta* | *chirurgica* | -122.21 | 46.1 | UW - Burke Museum | ice cave |
| *Grylloblatta* | *chirurgica* | -122.22 | 46.14 | UW - Burke Museum | ice cave |
| *Grylloblatta* | *chirurgica* | -122.22 | 46.13 | UW - Burke Museum | ice cave |
| *Grylloblatta* | *chirurgica* | -122.22 | 46.17 | UW - Burke Museum | ice cave |
| *Grylloblatta* | sp. | -122.23 | 46.18 | UW - Burke Museum | mountain |
| *Grylloblatta* | sp. | -121.51 | 46.87 | UW - Burke Museum | mountain |
| *Grylloblatta* | *campodeiformis* | -111.22 | 45.1 | Visscher *et al.*, 1982 | mountain |
| *Galloisiana* | *sinensis* | 128.05 | 42.03 | IZAS (Shu-Yong Wang) | mountain |
| *Grylloblattella* | *cheni* | 87.49 | 49.04 | IZAS (Ke-Qing Song) | mountain |
| *Galloisiana* | *chujoi* | 134.05 | 34.37 | Gurney, 1961 | ice cave |
| *Galloisiana* | *nipponensis* | 139.07 | 35.97 | Gurney, 1948 | mountain |
| *Galloisiana* | *nipponensis* | 135.83 | 34.88 | Gurney, 1948 | mountain |
| *Galloisiana* | *notabilis* | 129.83 | 32.78 | Storozhenko, 1996b | mountain |
| *Galloisiana* | *nipponensis* | 138.83 | 35.75 | Gokan *et al.*, 1982 | ice cave |
| *Grylloblattina* | sp. | 132.78 | 43.1 | Jarvis & Whiting, 2006 | mountain |
